# Size Sorting of Exosomes by Tuning the Thicknesses of the Electric Double Layers on a Micro-Nanofluidic Device

**DOI:** 10.3390/mi11050458

**Published:** 2020-04-28

**Authors:** Satoko Fujiwara, Kyojiro Morikawa, Tatsuro Endo, Hideaki Hisamoto, Kenji Sueyoshi

**Affiliations:** 1 Department of Applied Chemistry, Graduate School of Engineering, Osaka Prefecture University, Osaka 599-8531, Japan; szb02114@edu.osakafu-u.ac.jp (S.F.); endo@chem.osakafu-u.ac.jp (T.E.); hisamoto@chem.osakafu-u.ac.jp (H.H.); 2 Department of Applied Chemistry, School of Engineering, University of Tokyo, Tokyo 113-0033, Japan; morikawa@icl.t.u-tokyo.ac.jp; 3 Japan Science and Technology Agency (JST), Precursory Research for Embryonic Science and Technology (PRESTO), Tokyo 102-8666, Japan

**Keywords:** exosomes, size sorting, electric double layers, nanochannels, micro-nanofluidic device

## Abstract

Exosomes, a type of extracellular vesicle with a diameter of 30–150 nm, perform key biological functions such as intercellular communication. Recently, size sorting of exosomes has received increasing attention in order to clarify the correlation between their size and components. However, such sorting remains extremely difficult. Here, we propose to sort their size by controlling their electrokinetic migration in nanochannels in a micro-nanofluidic device, which is achieved by tuning the thickness of the electric double layers in the nanochannels. This approach was demonstrated experimentally for exosomes smaller than 250 nm. Using different running buffer concentrations (1 × 10^−3^, 1 × 10^−4^, and 1 × 10^−5^ M), most of the exosomes larger than 140, 110, and 80 nm were successfully cut off at the downstream of the nanochannels, respectively. Therefore, it is clarified that the proposed method is applicable for the size sorting of exosomes.

## 1. Introduction

Extracellular vesicles (EVs) play a significant role in the control of cellular and biological functions through multiple dynamic mechanisms. Exosomes are lipid membrane EVs of endocytic origin with a diameter of 30–150 nm. They are present in various body fluids, including blood, urine, and saliva, and contain biogenic compounds such as proteins, nucleic acids, and lipids. The components of exosomes depend on the type of cell, the cellular conditions, and the environment. Exosomes are important physiologically and pathologically, because they can mediate intercellular communication [1,2,3,4,5,6] such as that related to the metastasis of cancer [7,8,9]. Analyzing the constituents of exosomes may help identify the cells they are secreted from and understand their formation mechanism. Generally, EVs may be categorized into exosomes, microvesicles, and apoptotic bodies according to their size, composition, and biogenesis pathway. However, those divisions are not clear-cut, and their heterogeneity prevents the study of their biogenesis and action mechanisms without further separation [10]. Recently, the correlation between the size and components of exosomes has been studied to understand EVs including exosome biogenesis [11,12]. To examine this correlation, the exosomes have to be classified by size in increments of tens of nanometers. Various methods have been developed to separate and classify nanoparticles (NPs) smaller than 100 nm including the exosomes. Conventional methods such as ultracentrifugation and size-exclusion chromatography (SEC) [13,14,15,16,17] could easily isolate exosomes within a wide size range (30–150 nm) from other EVs. Nevertheless, these exosomes could not be further “sorted” or “classified” by their size, because of the random pore size in the columns for SEC. Other size-sorting methods have been proposed by using elaborate and highly regulated nanostructures integrated into micro-nanofluidic devices, due to the recent dramatic advancement in micro- and nano-electromechanical systems (MEMS and NEMS) [18,19,20,21,22,23,24]. Deterministic lateral displacement (DLD) is a popular method, and it works by using micro- and nano-pillar arrays tilted at an angle to generate unique flow streamlines. Wunsch et al. claimed to separate NPs with diameters of 20, 50, and 110 nm using nano-DLD at a low Péclet number. They also achieved the size-selective separation of exosomes, although the size distribution did not change a lot afterward [25]. Hattori et al. successfully separated 50 and 200 nm NPs using micro- and nano-pillar chips on an electroosmotic flow (EOF)-driven DLD, and also roughly separated EVs below and above 400 nm in size. Similarly, those authors could not change the size distribution of the exosomes so much before and after sorting, either [26]. Zeming et al. also reported the separation of 51 and 190 nm NPs using DLD devices under different ionic concentrations. Their device could separate 51–1500 nm NPs, with the disadvantages being the requirements for complex and extremely fine nano-structures and a long effective length. Further, this device failed to separate NPs below 100 nm (i.e., the size range of exosomes) [27]. Overall, reported DLD devices had difficulty separating NPs below 100 nm including exosomes, whereas they could separate NPs and submicron particles with high throughput. Furthermore, those devices often require regulated high pressure (~kPa), and they face some challenges in separating particles with diameters comparable to the critical diameter, which determines the threshold of the diameter values that could separate the NPs, of the device under the experimental conditions.

On the other hand, some nanofluidic devices have been successfully used to separate small particles and molecules such as NPs, DNA fragments, and proteins [28,29]. Wang et al. reported the selective preconcentration of proteins and peptides by electrostatic sieving in a nanochannel with a depth of 40 nm [28]. Under the experimental condition, the electric double layers (EDLs) on the upper-side and bottom-side inner surfaces of the nanochannel overlapped, which interfered with the transportation of large, negatively charged molecules such as proteins and peptides in the nanochannel by electrostatic repulsion. Fu et al. also reported the separation of DNA and proteins by the sieving effect of nanochannels. Nevertheless, there were some difficulties, i.e., smaller size of nanochannels than that of EVs, extending this separation method to NPs, especially in exosomes. Then, Regtmeier et al. demonstrated the continuous-flow separation of NPs based on electrostatic sieving at the micro-nanofluidic interface [30]. They managed to separate 15 and 39 nm NPs using a 525 nm deep nanochannel with weakly overlapped EDLs, although a detailed mechanism, including the correlation between the thicknesses of EDLs and separable sizes of NPs, was not reported. Consequently, the complete size sorting of NPs between 10 and 100 nm has not been achieved by nanofluidic devices utilizing electrostatic sieving from overlapped EDLs.

Here, we propose a size sorting method to tune the thicknesses of the EDLs (several to ca. 100 nm) in the nanochannels on a micro-nanofluidic device by controlling the electrolyte concentration. This method allows the classification of NPs including exosomes by size with an increment of tens of nanometers. Then, we demonstrated the size sorting for exosomes, and examined the correlation between the thicknesses of EDLs, the NP size, and the electrolyte concentration.

## 2. Theory

**Figure 1:**
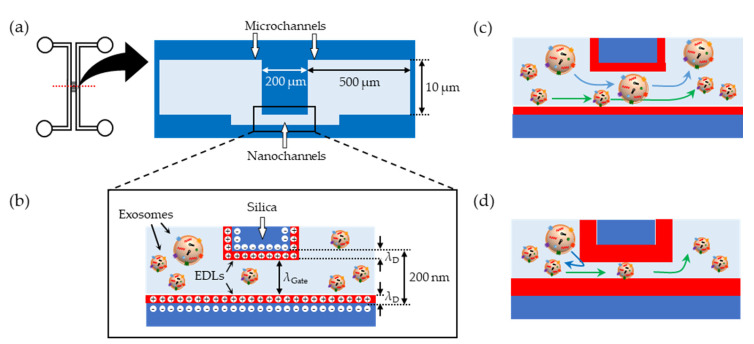
Schematic illustration of the proposed size sorting of exosomes. (**a**) Channel configuration and cross-sectional view of the developed micro-nanofluidic device. (**b**) Schematic of electric double layers (EDLs) formed on the inner surface of the nanochannels. (**c**,**d**) Schematic illustrations of the size sorting of exosomes by tuning the thicknesses of EDLs by employing background electrolytes at (**c**) high and (**d**) low concentrations.

In the silica-based micro-nanofluidic device developed herein, EDLs are formed on the negatively charged inner surfaces of the micro- and nanochannels because of the electrostatic repulsion between the anions in the running buffer and silanol groups on the surfaces (Figure 1a,b). The thickness of the EDL (Debye length, *λ*_D_ in units of nm) is estimated using Equation (1) [31],

(1) $$\lambda_{D\;}=\frac{9.6}{\sqrt{c}z}$$

where *c* is the concentration of the electrolytes in mM and *z* is the valency of the ionic species.

Equation (1) clearly shows that *λ*_D_ can be controlled from several to 100 nm by varying the electrolyte concentration. This, in turn, allows one to adjust the nanogate size (*λ*_Gate_ in units of nm), which is the space in the nanochannels through which the negatively charged exosomes [32] can pass (Figure 1b). At low electrolyte concentrations, the EDLs are thick and *λ*_D_ is large. Therefore, only smaller exosomes with a size less than *λ*_Gate_ can pass through the nanochannel by EOF, whereas those larger than *λ*_Gate_ cannot, as the EDLs prevent them from approaching nanochannels due to their electrostatic repulsion. (Figure 1c,d).

In this paper, the values of λ_Gate_ can be calculated by Equation (2) (*λ*_Gate,cal_) due to using 200 nm deep nanochannels.

(2) $$\lambda_{\mathrm{Gate},\mathrm{cal}}{=200\;-\;2\lambda }_{D}$$

According to Equations (1) and (2), *λ*_Gate,cal_ can also be controlled by tuning the electrolyte concentration, which allows the sorting of exosomes according to their size (Figure 1c,d). Figure S1 also shows the variation in *λ*_D_ and *λ*_Gate,cal_ with 2-(4-(2-hydroxyethyl)-1-piperazinyl)ethane sulfonic acid (HEPES) concentrations.

## 3. Materials and Methods

### 3.1. Materials

The fused-silica plates (VIOSIL-SX) were purchased from Shin-Etsu Quartz Co., Ltd., (Tokyo, Japan) for fabrication of the micro-nanofluidic device. A ZEP-520A used as an electron beam resist for fabrication of the nanochannels was purchased from Zeon Corp. (Tokyo, Japan). A KMPR^®^ 1035 used as a photoresist in the photolithography for fabrication of the microchannels was bought from Microchem Corporation (Westborough, MA, USA). To fabricate the additional reservoirs on the micro-nanofluidic device, the prepolymer polydimethylsiloxane (PDMS, SILPOT 184) and curing agent (SILPOT 184 CAT) were purchased from Dow Corning Toray Co., Ltd. (Tokyo, Japan). Exosome samples were derived from the HEK293 human embryonic kidney cell line (EXOP-110A-1). Exo-Red and ExoQuick-TC for labelling exosomes were purchased from System Biosciences (Palo Alto, CA, USA). Negatively charged fluorescent NPs (micromer^®^-greenF and sicaster^®^-greenF; Nacalai Tesque, Inc., Kyoto, Japan) were used to preliminarily confirm and evaluate the proposed method (Tables S1 and S2). HEPES and sodium hydroxide (NaOH) were bought from Wako Pure Chemical Industries Co. (Osaka, Japan) to prepare the running buffers used for the size sorting of exosomes and NPs. The phosphate buffered saline (PBS) was prepared using disodium hydrogen phosphate 12-hydrate (Na_2_HPO_4_·12H_2_O), potassium chloride (KCl), and potassium dihydrogenphosphate (KH_2_PO_4_), all from Wako Pure Chemical Industries Co. (Osaka, Japan), as well as sodium chloride (NaCl; Kanto Chemical Co. Inc., Tokyo, Japan). Ultrapure deionized water with a resistance of 1.8 × 10^7^ Ω cm at 25 °C was used to prepare the HEPES and 1×PBS buffers.

### 3.2. Apparatus

To fabricate the micro-nanofluidic device, electron beam lithography (ELS-7500; Elionix, Tokyo, Japan) and the dry etching apparatus (NLD-550; ULVAC Co., Ltd., Kanagawa, Japan) were used. A pH meter (F-72; HORIBA, Kyoto, Japan) and an ultrapure water system (Direct-Q UV3; Merck, Tokyo, Japan) were used to prepare the HEPES and PBS buffers. A digital microscope and a fluorescence microscope equipped with a CCD camera (VHX-900 and VB-7010, respectively; KEYENCE, Osaka, Japan) were used to observe the fluorescence. The constant voltage applied to the sorting device came from a high-voltage power supply (HVS448LC-6000D; LabSmith, CA, USA). A UV/O_3_ apparatus (Filgen, Aichi, Japan) was used for bonding the silica device and the PDMS reservoir. The particle sizes before and after the proposed sorting were measured using qNano (IZON Science Ltd., Christchurch, New Zealand). A dynamic light scattering (DLS) apparatus (ELSZ-DN2; Otsuka Electronics Co., Ltd., Osaka, Japan) was used to measure the sizes of fluorescent NPs (Figures S2–S5). A tube rotator (TR-350, AS ONE Co., Ltd., Osaka, Japan) and aluminum block bath with a cool-thermo unit (CTU-mini, TAITEC Co., Ltd., Saitama, Japan) were employed for labeling exosomes

### 3.3. Fabrication of the Micro-Nanofluidic Device

**Figure 2:**
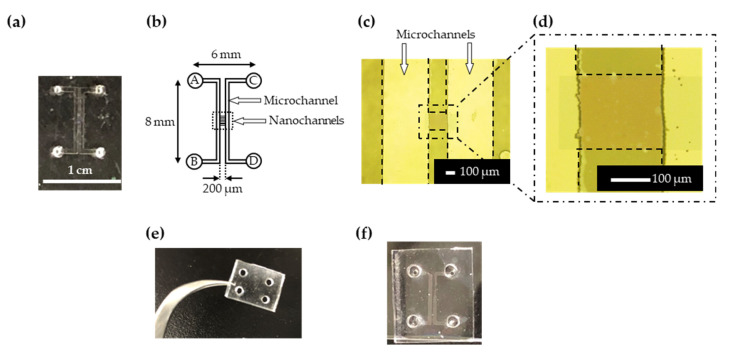
(**a**) Photograph and (**b**) channel configuration of the micro-nanofluidic device. (**c**) Microscopic images of the microchannels near the nanochannels. Black dashed lines represent the microchannels and nanochannels. (**d**) Enlarged image of the nanochannels. Photographs of (**e**) the PDMS reservoirs and (**f**) the device bonded with reservoirs.

To confirm and evaluate the size sorting of exosomes, the micro-nanofluidic device (Figure 2a,b) was fabricated by standard electron-beam lithography, photolithography, dry etching, and bonding techniques [33,34,35].

The nanochannels (width: 1 μm, depth: 200 nm, channel distance: 1 μm) were fabricated on a fused-silica plate via a combination of electron beam lithography and dry etching. In the electron beam lithography, a ZEP-520A was used as an electron beam resist. The dry etching using an NLD-550 was performed using a mixture of CHF_3_ and SF_6_ gases. The depth of the nanochannels was 200 nm because the tunable region of EDLs was below approximately 100 nm.

Two U-shaped sample injection microchannels (width: 500 μm, depth: 10 μm) were fabricated on another substrate using photolithography and dry etching processes. KMPR^®^ 1035 was used as a photoresist in the photolithography, and dry etching was performed using a mixture of C_3_F_8_, CHF_3_, and Ar gases. The U-shaped microchannels were located on both sides of the nanochannels. Inlet holes (Figure 2b, A–D) were bored through the patterned substrates using a diamond-coated drill.

The micro/nano-fabricated plates were then bonded. Before the bonding, both plates were washed with a mixed three-to-one solution of sulfuric acid and hydrogen peroxide. Then the micro/nano-fabricated plate was ultra-sonicated for 10 min. After activating the surface of glass plates with oxygen and fluorine plasma, the plates were bonded at a temperature of 110 °C for five hours (Figure 2a–d).

Additional reservoirs made of PDMS (Figure 2e) were attached to this device by surface activation using the UV/O_3_ treatment (Figure 2f).

### 3.4. Methods

#### 3.4.1. Fluorescence Labeling of Exosomes

Before their electrokinetic migration, the exosomes were labelled to facilitate their observation under a fluorescence microscope. To this end, an exosome dispersion was prepared by adding 50 μL of 10× Exo-Red to 500 μL of resuspended exosome in 1×PBS (pH 7.4) (whose details are 15 μL of exosomes and 485 μL of 1×PBS). The dispersion was mixed well by flicking or inversion and then incubated at 37 °C for 10 min. To stop the labeling reaction, 100 μL of ExoQuick-TC reagent was added to the suspension and then mixed by inverting 6 times. The labeled exosome sample was put on ice for 30 min. After centrifugation for 3 min at 14,000 rpm in a microcentrifuge, the supernatant with excess labeling reagent was removed, and the labeled exosome pellet was suspended in 500 μL of 1×PBS.

#### 3.4.2. Electrokinetic Migration of Exosomes

**Figure 3:**
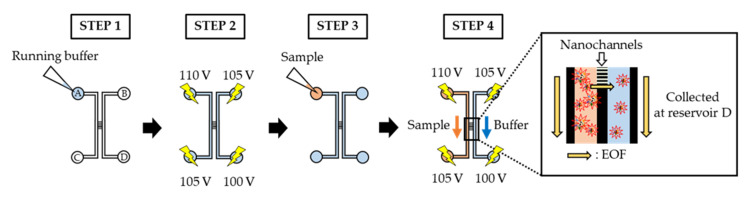
Protocols for the size sorting of exosomes.

First, the micro-nanofluidic channels and reservoirs were filled with the running buffer (HEPES, pH 7.4, 1 × 10^−5^ to 1 × 10^−3^ M) (Figure 3, STEP 1). The device was then washed by the EOF for 30 min (applied voltages on the reservoirs, A: 110 V, B: 105 V, C: 105 V, D: 100 V) (STEP 2). After replacing the running buffer in the device with a fresh one, 7 μL of the sample dispersion was placed in reservoir A (STEP 3). Finally, each sample dispersion was electrokinetically introduced into the device (A: 110 V, B: 105 V, C: 105 V, D: 100 V) and allowed to remain for 60 min (STEP 4). After employing each running buffer (Table 1), the exosomes reaching reservoir D by electrokinetic migration were collected. These protocols were repeated thrice to gather enough exosome dispersions for size measurement.

#### 3.4.3. Size Evaluation of Collected Exosomes

The size distribution of exosomes collected at reservoir D was measured by qNano, which uses tunable resistive pulse sensing (TRPS) based on the Coulter principle at the nanoscale [36,37]. Table 1 also shows the conditions of each size measurement.

## 4. Results

**Figure 4:**
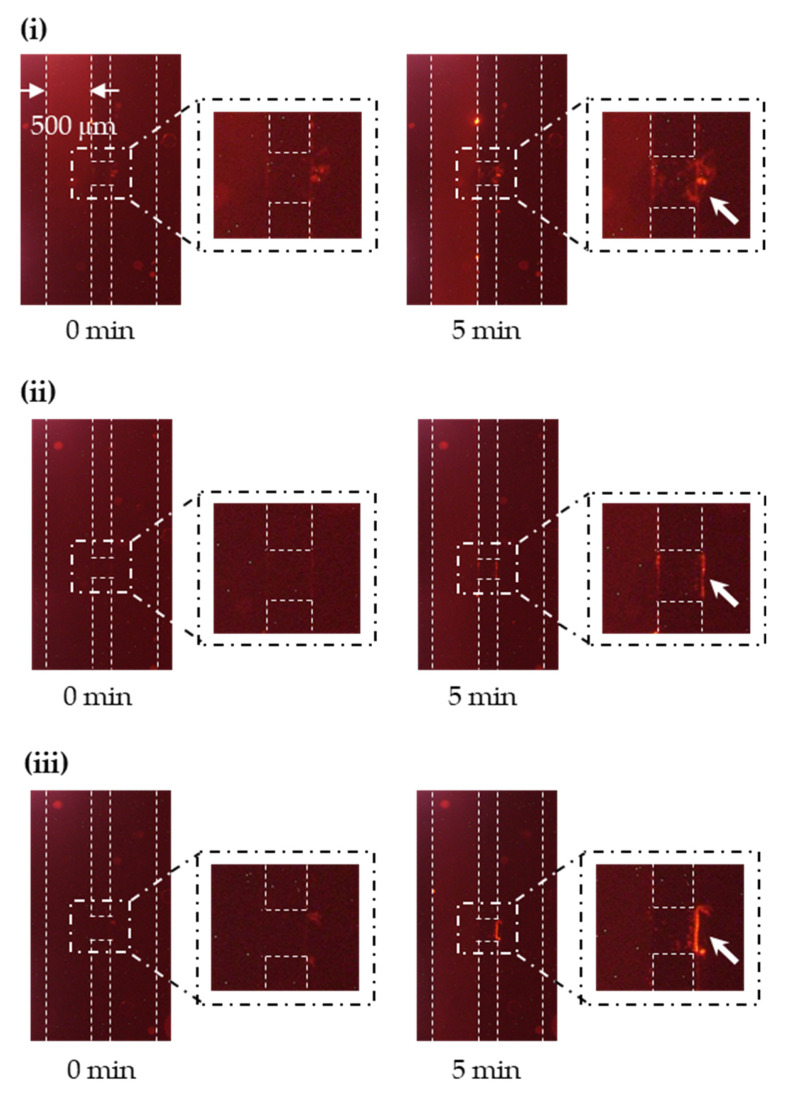
Fluorescence images during the size sorting of exosomes under each experimental condition. White dashed lines denote the microchannels and nanochannels, while white dot-dashed lines enclose the enlarged view around the nanochannels. White arrows indicate that the exosomes can pass through the nanochannels within 5 min. The concentration of the running buffer is (**i**) 1 × 10^−3^, (**ii**) 1 × 10^−4^, and (**iii**) 1 × 10^−5^ M 2-(4-(2-hydroxyethyl)-1-piperazinyl)ethane sulfonic acid (HEPES).

**Figure 5:**
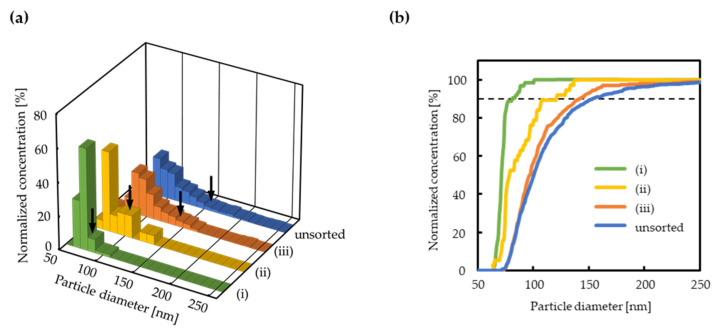
Results of the size sorting of exosomes. (**a**) Distribution of exosome sizes before and after sorting. The black arrows indicate *d*_90_ in each distribution. (**b**) Cumulative distribution of exosome size. Black dashed line: *d*_90_. The concentrations of the running buffer under (i)–(iii) are the same as those in Figure 4.

Before applying the proposed size sorting to exosomes, preliminary experiments as a proof of concept were carried out using fluorescent NPs. The data are shown in (Supplementary Materials Figures S6 and S7). When using the exosomes, the fluorescence images show that the exosomes were successfully transferred through the nanochannels under each experimental condition, because fluorescence appeared near the downstream of the nanochannels (Figure 4). After applying the voltages, the running buffer in reservoir D (which contained exosomes that passed through the nanochannels) was collected in triplicates. qNano was used to evaluate the size distribution of exosomes before and after the size sorting. The results (Figure 5a, Figure S8) show that the size distribution of collected exosomes became narrower when decreasing the concentration of the running buffer. Specifically, the value of *d*_90_ (the particle size corresponding to 90% of the cumulative distribution) became drastically smaller under conditions (iii), (ii), and (i) compared to that of the “unsorted” exosomes. Most of the exosomes larger than 140, 110, and 80 nm were also successfully cut off under (iii), (ii), and (i), respectively (Figure 5b, Table 2). The values of *d*_90_ and *d*_max_ (the maximum particle size in the collected sample) are roughly consistent with *λ*_Gate,exp_, which is the *λ*_Gate_ value estimated from preliminary experiments using fluorescent NPs (Tables S3–S7). On the other hand, the total concentration of the unsorted exosome dispersion (6.22 × 10^10^ particles/mL) decreased to 2.46 × 10^8^–1.65 × 10^9^ after sorting (Figure S8). The main reason was that only a small amount of exosomes migrating nearby the nanochannels could pass through there and reach the collection reservoir, resulting in both the low throughput of sorting and the decrease in the total concentration of the collected dispersion. However, we do not think that the low throughput is not the critical issue in the fundamental confirmation. We could collect enough concentration of sorted exosomes by employing this device for the proof of the proposed concept, although a long operation time was required (60 min, three times).

In experimental condition (i), *λ*_Gate,cal_ is 8 nm, whereas *d*_max_ and *λ*_Gate,exp_ are very different (102 and 70–140 nm, respectively). However, it is reasonable that *λ*_Gate,cal_ is not consistent with *λ*_Gate,exp_, because the EDL overlap does not signify complete NP impermeability of the nanochannel as previously reported [38,39]. This inconsistency also occurred in our preliminary experiment using the fluorescence NPs (see supplementary materials), and it could be caused by two specific nanoscale phenomena. The first is the excess protons induced by proton exchange between the charged surface with ionizable silanol groups (SiOH) and the water molecules, as shown in Equation (3) [38,40,41,42].

(3) $$\left(\equiv {\;\mathrm{SiOH}+H}_{2}O\right){+H}_{2}O\;↔{\;\mathrm{SiO}}^{-}+\left(H_{3}O^{+}{+H}_{2}O\right)\to {\;\mathrm{SiO}}^{-}{+(H}_{2}{O+H}_{3}O^{+})$$

Secondly, surfaces of the exosomes [32] and fluorescent NPs modified by the carboxyl group acquire a negative charge at pH 7.4, and, as a result, the exosomes were surrounded by their counter cations and moved along with them in the device. Consequently, the local ionic strength (*I*) in the nanochannels increased because of both phenomena mentioned above, which directly contributed to *λ*_D,exp_ owing to the relation between *I* and *c* (Equation (4)) and Equation (1). Hence, *λ*_Gate,exp_ could be larger than *λ*_Gate,cal_ shown in Tables 2 and S7 when the electrolyte solution was extremely diluted.

(4) $$I=\frac{1}{2}\;\underset{i}{\sum }c_{i}z_{i}^{2}$$

Our experimental results successfully demonstrated the proposed size sorting method by tuning the thicknesses of the EDLs. Moreover, the correlation among *λ*_D_, the electrolyte concentration (*c*), and the size of sorted NPs or exosomes could also be confirmed by using the highly regulated micro-nanofluidic device made from quartz. Furthermore, the method was able to sort exosomes more finely, with size increments of approximately 30 nm. Compared to previous reports using micro- and nano-DLD arrays [25,26], our simple micro-nanofluidic device achieved drastic changes in both the size distribution and *d*_max_ values. Smith et al. developed integrated nanoscale DLD arrays and demonstrated the separation of exosomes from urine and serum with high throughput. However, their *d*_max_ did not change so much for both Bump and ZigZag flow, and the size distribution after sorting was still wide [43]. Nevertheless, the throughput of the present device was much lower than that in the DLD devices, mainly because the former was fabricated just to confirm the concept. To realize high throughput for exosomes using our method, more detailed investigations are required such as better device design based on hydrodynamics simulation and increasing the number of nanochannels. Although EOF is a dominant factor in the transport of exosomes, it was not discussed in the present study, as its precise evaluation calls for three-dimensional simulation of the electric fields in the micro-nanofluidic device. Additionally, the strong electric field in the nanochannels (estimated to be approximately 100 V/cm) may damage the structure/stability of exosomes [26,44]. Therefore, future studies also have to consider the effects of the applied electric field on the EOF and exosome structure/stability during the size sorting.

## 5. Conclusions

In this work, we proposed a size sorting method for exosomes by tuning the thicknesses of EDLs in the nanochannels in a micro-nanofluidic device, and this method allowed the classification of exosomes by size with an increment of tens of nanometers. For exosomes that passed through the device, their size distribution and *d*_90_ became respectively narrower and smaller upon decreasing the running buffer concentration, indicating that the exosomes were successfully sorted by adjusting only the electrolyte concentration. It was also confirmed that *d*_90_ and *d*_max_ are roughly consistent with the value of *λ*_Gate,exp_, also suggesting that the size sorting occurred by tuning the thicknesses of the EDLs. Moreover, a correlation among *λ*_D_, the electrolyte concentration, and the exosome size was confirmed. Improvement in the device throughput by changing its configuration with theoretical simulations and further examinations of this correlation will lead to applications in the size sorting of exosomes. To realize a further improved sorting device, a new micro-nanofluidic device equipping a sheath-flow channel and additional nanochannels was already fabricated. Fundamental evaluations of sorting performance of the newly fabricated device and its application are in progress.

## Figures and Tables

**Table 1:** Experimental conditions of the electrokinetic transportation of exosomes, and the size measurement condition.

Experimental Condition (Running Buffer Used)	Measurement Condition	
	Dilution Buffer	Rate of Dilution
(i) 1 × 10^−5^ M HEPES *	2×PBS	× 5–100
(ii) 1 × 10^−4^ M HEPES	1×PBS	
(iii) 1 × 10^−3^ M HEPES		
unsorted **		

* 2-(4-(2-hydroxyethyl)-1-piperazinyl)ethane sulfonic acid, ** Without size sorting.

**Table 2:** *d*_90_ and *d*_max_ values in the collected exosomes, and *λ*_Gate,cal_ and *λ*_Gate,exp_ values under each condition.

Size (nm)		Experimental Condition			
		(i) 1 × 10^−5^ M HEPES	(ii) 1 × 10^−4^ M HEPES	(iii) 1 × 10^−3^ M HEPES	Unsorted
Exosomes	*d*_90_	81	112	142	153
	*d*_max_	102	138	180 *	434
Nanogates	*λ*_Gate,cal_	8	139	181	-
	*λ*_Gate,exp_	70–140		-	

* The large particles considered to be aggregates were excluded. Please see Figure S8 for details.

## Supplementary Materials

The following are available online at https://www.mdpi.com/2072-666X/11/5/458/s1, Figure S1: The correlation between *λ*_D_ and *λ*_Gate,cal_ under various HEPES concentrations, Figures S2–S5: Size distribution of 140, 70, and 40 nm particles, Figure S6: Protocols for the size sorting of fluorescent NPs, Figure S7: Schematic illustrations of analysis protocols of the fluorescence images; Figure S8: Size distributions of collected exosome samples under each experimental condition, Table S1: Particle diameter (*d*_z_) of the fluorescent NPs measured by DLS, Table S2: Properties of the fluorescent NPs, Tables S3–S6: Results of electrokinetic migration for the 140, 70, and 40 nm particles, Table S7: Evaluations of electrokinetic migrations of each NP by tuning the HEPES concentrations.
