# Supplementary material for: Size Sorting of Exosomes by Tuning the Thicknesses of the Electric Double Layers on a Micro-Nanofluidic Device
Source: Micromachines (Basel). 2020 Apr 28;11(5):458. doi: 10.3390/mi11050458 (PMC7281254; doi:10.3390/mi11050458)
Supplement: Supplementary file 1 [file micromachines-11-00458-s001.pdf]

micromachines

MDPI

Supplementary Materials

Size Sorting of Exosomes by Tuning the Thicknesses of the Electric Double Layers on a Micro-Nanofluidic Device

Satoko Fujiwara, Kyojiro Morikawa, Tatsuro Endo, Hideaki Hisamoto and Kenji Sueyoshi *

S1. The Correlation between λ_D and $\lambda_{Gate,cal}$

Figure S1 shows the variation in λ_D and $\lambda_{Gate,cal}$ with 2-(4-(2-hydroxyethyl)-1-piperazinyl)ethane sulfonic acid (HEPES) concentrations.

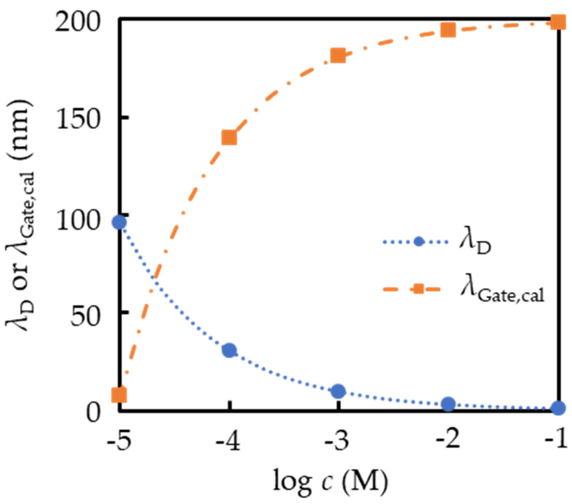

Figure S1. The correlation between λ_D and $\lambda_{Gate,cal}$ under various 2-(4-(2-hydroxyethyl)-1-piperazinyl)ethane sulfonic acid (HEPES) concentrations (*c*).

S2. Properties of the Fluorescent NPs

Table S1 and Figures S2–S5 show the measured particle diameter (d_z) and the size distributions of the fluorescent NP dispersions under different buffer concentrations (a–c) measured by DLS, respectively.

Table 1. Particle diameter (d_z) of the fluorescent NPs measured by dynamic light scattering (DLS).

Product name	d_{NP} (nm)	d_z (nm) (a) 1 × 10⁻⁵ M HEPES*	(b) 1 × 10⁻⁴ M HEPES	(c) 1 × 10⁻³ M HEPES
micromer®-greenF	140	148.4	144.6	141.4
	70	67.3	65.4	62.5
	40	43.4	40.5	43
sicaster®-greenF	70	61.6	63.1	61.6

* 2-(4-(2-hydroxyethyl)-1-piperazinyl)ethane sulfonic acid

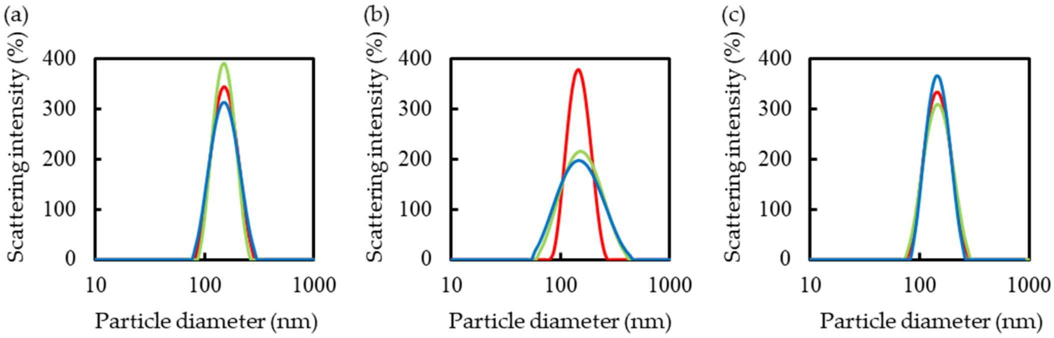

Figure S2. Size distribution of 140 nm particles.

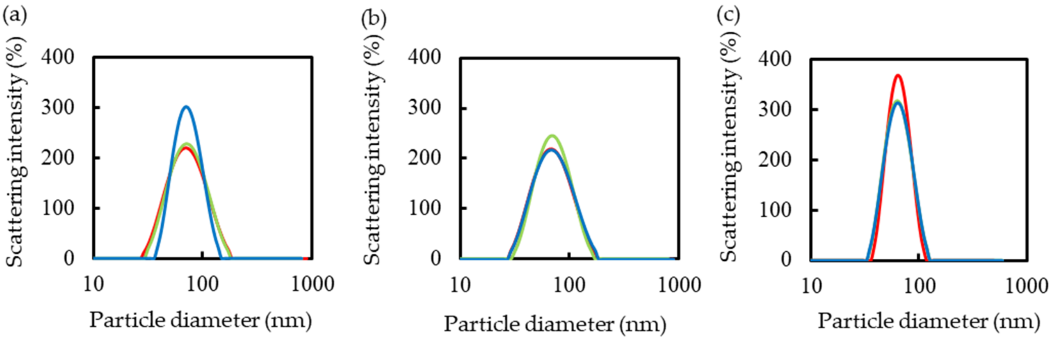

Figure S3. Size distribution of 70 nm particles (micromer®-greenF).

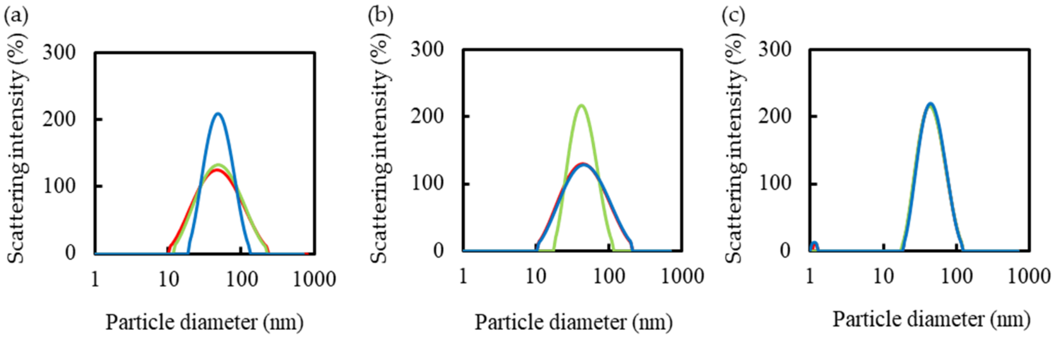

Figure S4. Size distribution of 40 nm particles.

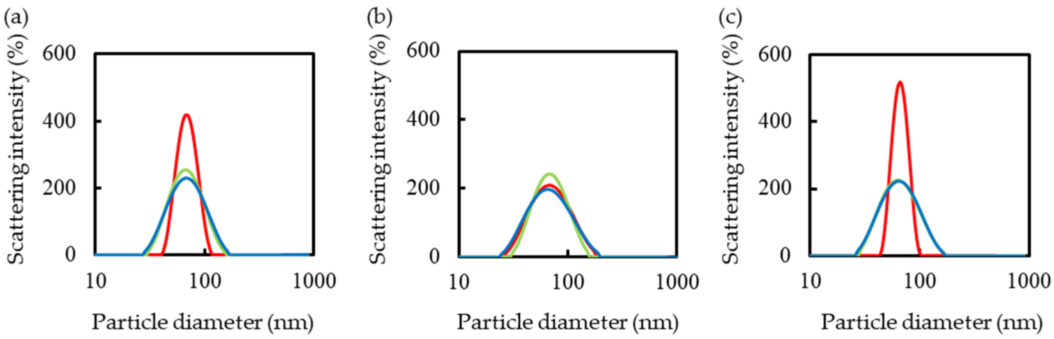

Figure S5. Size distribution of 70 nm particles (sicaster®-greenF).

Table S2 shows the properties of the fluorescent NPs. In the experiments, micromer®-greenF NPs were used. On the other hand, sicaster®-greenF NPs were used to confirm whether the results obtained by micromer®-greenF NPs were correct.

Table S2. Properties of the fluorescent NPs.

Product name	d_{NP} (nm)	Composition	Surface charge density (µmol/g)
micromer®-greenF	140	green fluorescent polystyrene	4
	70		8
	40	green fluorescent polymethacrylate	10
sicaster®-greenF	70	green fluorescent silica	1

S3. Size Sorting of the Fluorescent NPs

S3.1. Electrokinetic Migration of the Fluorescent NPs

First, the micro-nanofluidic device was filled with the running buffer (1×10^{-6}–1×10^{-2} M, pH 7.4) (Figure S6; STEP 1). The device was then washed by electrophoresis for 30 min (applied voltages; A: 110 V, B: 105 V, C: 105 V, D: 100 V) (Figure S6; STEP 2). After replacing the running buffer in the device with a fresh one, 2 µL of each sample dispersion and 4 µL of the running buffer were placed in reservoir A (Figure S6; STEP 3). Finally, each sample dispersion was electrokinetically introduced into the device (A: 110 V, B: 105 V, C: 105 V, D: 100 V), and the electrokinetic migration of the NPs via nanochannels was observed using a fluorescence microscope (Figure S6; STEP 4).

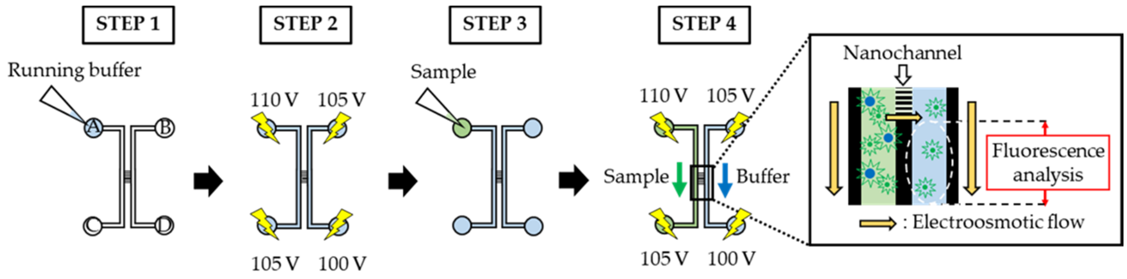

Figure S6. Protocols for the size sorting of fluorescent NPs.

S3.2. Results and Discussion of the Size Sorting of NPs

S3.2.1. Analysis of the Obtained Fluorescence Images for Size Sorting the Fluorescent NPs

First, the fluorescence profiles of each obtained fluorescence image at three different points of the upside and downside of the nanochannels (line 1 and 2 in Figure S7a,b) were obtained. Then, the difference between the fluorescence intensity of line 1 and 2 (i.e., line 2 minus line 1) was calculated and a new profile was obtained (Figure S7c). Finally, the integration value of the fluorescence at the right microchannel, which was shown as the value of red flame "28" in Figure S7c, was calculated and compared to 3 times the standard deviation (3σ) of the background fluorescence intensity.

For example, in the case of 140 nm particles, each integration value (*Ψ*) was 198 and −426 to 3*σ* (= 9) under 1 × 10^{-2} and 1 × 10^{-4} M HEPES buffer, respectively. Therefore, 140 nm particles could be passed through nanochannels under 1 × 10^{-2} M HEPES buffer, whereas they could not under 1 × 10^{-4} M HEPES buffer (Figures S7d,e).

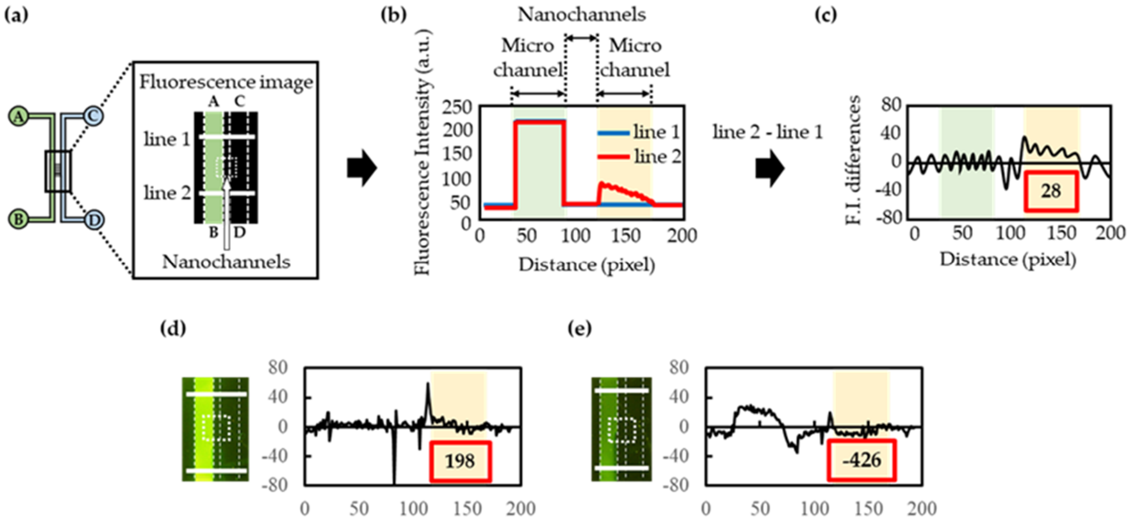

Figure S7. Schematic illustrations of analysis protocols of the fluorescence images.

In summary, Tables S3–S6 show the obtained fluorescent images, integration value (*Ψ*), and standard deviation (3*σ*) of the background fluorescence intensity under each NP size and HEPES concentration.

Table 3. Results of electrokinetic migration in the case of 140 nm particles.

HEPES Concentration [M]	1 × 10^{-5}	1 × 10^{-4}	1 × 10^{-3}	1 × 10^{-2}
Fluorescence image	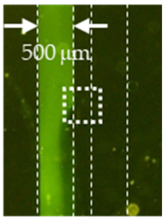	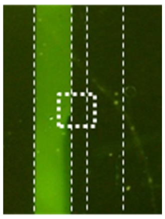	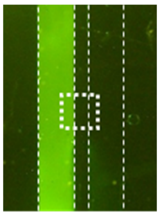	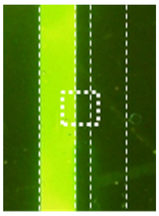
Ψ	2	−426	262	198
3*σ*	9	9	9	9

Table S4. Results of electrokinetic migration in the case of 70 nm particles (micromer®-greenF).

HEPES concentration (M)	1 × 10^{-5}	1 × 10^{-4}
Fluorescence image	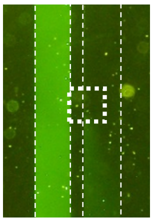	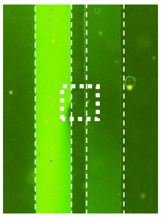
Ψ	845	509
3*σ*	9	16

Table S5. Results of electrokinetic migration in the case of 40 nm particles.

**HEPES concentration (M)**	**1 × 10^{-6}**	**1 × 10^{-5}**	**1 × 10^{-4}**
Fluorescence image	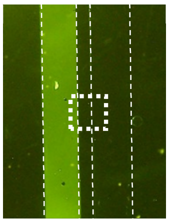	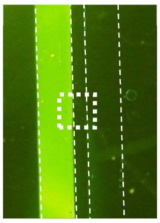	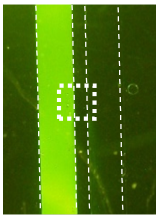
Ψ	–95	57	179
3σ		9	

Table S6. Results of electrokinetic migration in the case of 70 nm particles (sicaster®-greenF).

HEPES concentration (M)	1 × 10^{-5}	1 × 10^{-4}
Fluorescence image	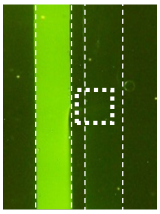	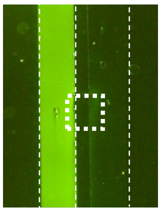
Ψ	544	297
3σ	9	16

S3.2.2. Evaluation of Size Sorting of the Fluorescent NPs

According to the analysis of the obtained fluorescence images, we evaluated whether NPs could pass through nanochannels under each HEPES concentration as the running buffers. Table S7 summarizes the results of the electrokinetic migration through nanochannels by tuning the concentration of the HEPES buffers, wherein $\lambda_{\mathrm{Gate,cal}}$ is calculated using Equations (1) and (2) and $\lambda_{\mathrm{Gate,exp}}$ is estimated from the electrokinetic migration of the fluorescent NPs via the nanogates. We observe that the values of $\lambda_{\mathrm{Gate,cal}}$ and $\lambda_{\mathrm{Gate,exp}}$ are consistent at concentrations of 1 × 10^{-4}–1 × 10^{-2} M, indicating that the correlation mostly followed the theory. However, at 1 × 10^{-5} M buffer concentration, 70 nm or smaller NPs could pass through, although $\lambda_{\mathrm{Gate,cal}}$ is only 8 nm; this could be caused by the specific nanoscale phenomena as previously mentioned in the article. Thus, $\lambda_{\mathrm{Gate,exp}}$ could be larger than 70 nm, whereas $\lambda_{\mathrm{Gate,cal}}$ under the 1 × 10^{-5} M HEPES buffer was ~10 nm. These results suggest that 40–140 nm NPs can be sorted by varying the running buffer concentration between 1 × 10^{-5} and 1 × 10^{-4} M. In addition, we confirmed the proposed concept, i.e., size sorting of NPs by tuning the thicknesses of the EDLs in the nanochannels using the developed micro-nanofluidic device was demonstrated.

According to these results from the preliminary NP experiments, we considered that the proposed size sorting method was applicable for exosomes.

Table S7. Evaluations of electrokinetic migrations of each NP by tuning HEPES concentrations.

		**HEPES Concentration (c) (M)**				
		**1×10^{-6}**	**1×10^{-5}**	**1×10^{-4}**	**1×10^{-3}**	**1×10^{-2}**
d_{NP} (nm)	140	No	No	No	Yes	Yes
	70	No	Yes	Yes	Yes	Yes
	40	No	Yes	Yes	Yes	Yes
$\lambda_{Gate,cal}$ (nm)		-*	8	139	181	197
$\lambda_{Gate,exp}$ (nm)		<40		70–140		>140

* Nanogate is closed by the overlap of the EDLs from Equations (1) and (2).

S4. Size Distribution of Size Sorting of Exosomes under Each Experimental Condition

Figure S8 shows the size distribution of obtained exosomes under each experimental condition on the device. However, in the case of (iii), a few numbers of exosomes ranging over 180 nm were observed (shown as * in Figure S8, d_{max}: 480 nm). This result suggested that these large particles were considered to be the aggregation of some exosomes as there were so few of them (2.5%/total) and they were far from the main distributions ranging below 180 nm. Therefore, these values were not important and they were excluded from the value of d_{max} listed in Table 2.

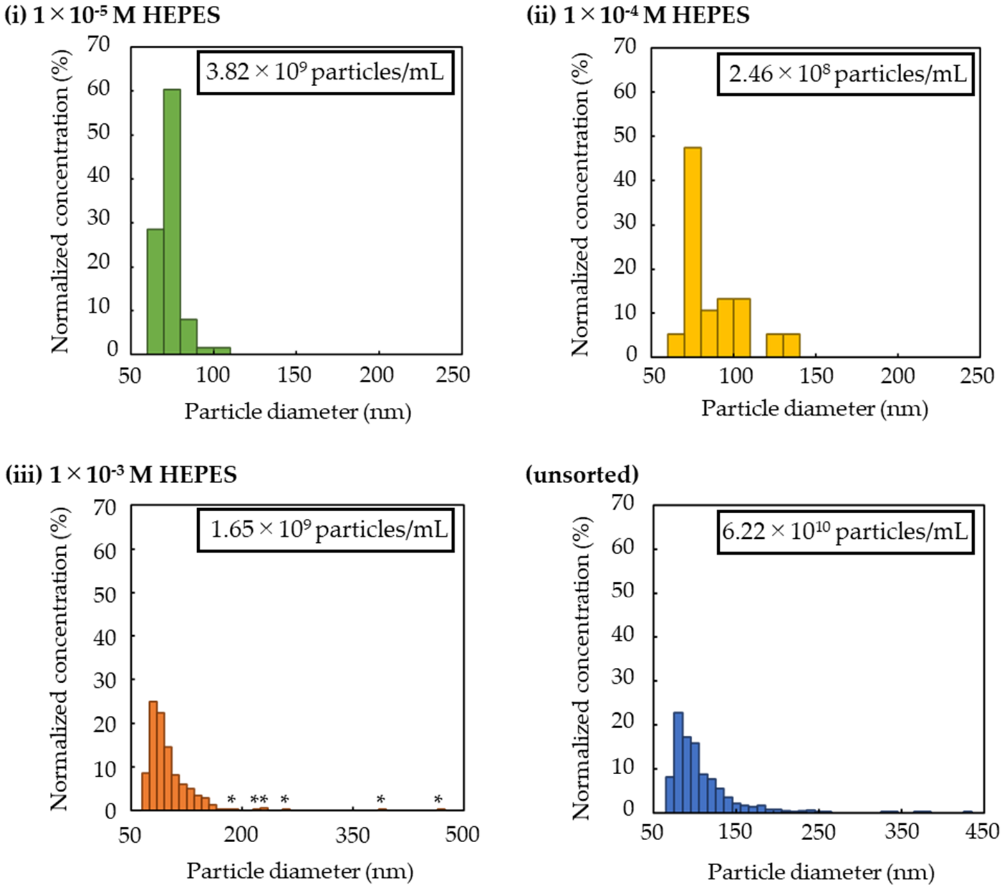

Figure S8. Size distributions of collected exosome samples under each experimental condition (values surrounded by black squares in each graph are raw concentrations).

© 2020 by the authors. Submitted for possible open access publication under the terms and conditions of the Creative Commons Attribution (CC BY) license (http://creativecommons.org/licenses/by/4.0/).
